# Sec10 suppresses antiviral innate immune response by facilitating STUB1-mediated STAT1 degradation

**DOI:** 10.1371/journal.ppat.1013472

**Published:** 2025-09-08

**Authors:** Fachao Sun, Wenqing Ma, Yanan Xu, Luteng He, Xiao Yu, Xingyu Li, Yingying Li, Daniel Chang He, Hongmei Wang, Hongbin He

**Affiliations:** 1 Ruminant Diseases Research Center, College of Life Sciences, Shandong Normal University, Jinan, Shandong, China; 2 Department of Preventive Veterinary Medicine, College of Veterinary Medicine, Shandong Agricultural University, Taian, Shandong, China; 3 The College of Arts and Sciences, University of North Carolina at Chapel Hill, Chapel Hill, North Carolina, United States of America; University of Alabama at Birmingham, UNITED STATES OF AMERICA

## Abstract

The exocyst complex is a heterooctameric protein complex, the individual components of the complex are thought to act on specific biological processes. However, the role of Sec10, the central subunit of the complex, in host defense and viral replication remains unclear. Here, we reported that Sec10 significantly impairs the activation of JAK-STAT signal pathway of type I IFN (IFN-I) response against both DNA- and RNA-viruses, and promotes viral replication, respectively. Mechanistically, Sec10 interacts with E3 ligase STUB1, promotes the interaction of STUB1 and STAT1, and consequently accelerate STUB1-mediated proteasomal degradation of STAT1 via K6-linked polyubiquitination at Lys240 and Lys652, thus weakens STAT1 triggered antiviral immune responses. More importantly, myeloid-specific deletion of Sec10 in mice showed enhanced IFN-I response against viral infection and improved survival of mice. Collectively, these findings demonstrate that Sec10 attenuates the JAK-STAT signaling pathway by targeting STAT1 for proteasomal degradation and identifies a previously unknown function of Sec10 in antiviral innate immunity and viral replication.

## Author summary

Type I interferon (IFN-I) signaling pathway plays an important role in host’s antiviral response. Host proteins are involved in regulating cellular processes to regulate various viral infections, including HSV-1 and VSV. The role of Sec10 as a central subunit of the complex in host defense and viral replication is unclear. Here, we reported that Sec10 significantly facilitated HSV-1 and VSV replication and inhibited the expression of IFN-β. We found that Sec10 negatively regulated IFN-I signaling pathway by targeting STAT1. We also found that Sec10 recruits STUB1 to catalyze K6-linked ubiquitination of STAT1 at K240 and K652, thereby blocking the production of ISGs and facilitating virus replication. Together, our study uncovers a novel mechanism for Sec10 to regulate HSV-1 and VSV infection.

## Introduction

The innate immune system serves as the first line of host defense viral infection. Upon viral infection, pattern recognition receptors (PRRs), including Toll-like receptors (TLRs), RIG-I-like receptors (RLRs), NOD-like receptors (NLRs), and cyclic guanosine monophosphate–adenosine monophosphate synthase (cGAS), surveil the pathogen-associated molecular patterns (PAMPs) and thereby trigger the activation of type 1 interferon (IFN-I) signaling [1,2]. The IFNs trigger the JAK/STAT signaling pathway by binding to the cognate IFN receptor (IFNAR). The IFNAR1 and IFNAR2 complex phosphorylate and activate Janus kinases JAK1 and tyrosine kinase 2 (Tyk2), respectively. Subsequently, the downstream signal transducer and activator of transcription STAT1 and STAT2 are phosphorylated, which forms the IFN-stimulated gene factor 3 (ISGF3) complexes with IFN regulatory factor 9 (IRF9). The ISGF3 complexes translocate to the nucleus and specifically bind to IFN response elements (ISREs), thereby activating the transcription of downstream IFN-stimulated genes (ISGs), which have a central role in intracellular antiviral defenses [3].

The signal transducer and activator of transcription 1 (STAT1) protein is critical in signal transducers of the cellular response to IFNs, a critical component of the antiviral innate immune response [4]. Upon IFNs stimulation or viral infection, IFN binds to cell-surface receptors, activating STAT1, leading to its phosphorylation, dimerization, and translocation to the nucleus, where it regulates gene expression and enhances the cell’s antiviral function [5]. Patients with STAT1 mutations have complete blockage of the IFN/STAT1 pathway and are susceptible to fatal bacterial and viral infections [6,7]. Viruses antagonize the IFN-induced host antiviral effects through targets STAT1 in different ways. For instance, our previous research found that bovine herpesvirus-1 UL41 abrogates the JAK-STAT-mediated signal pathway by binding and degrading STAT1 via its RNase activity [8,9]. Human metapneumovirus can inhibit the IFN-I response through preventing STAT1 phosphorylation and nuclear translocation, causing persistent lung infections and evading host clearance of the virus [10]. The ubiquitination of STAT1 is a post-translational modification process, which is crucial for the homeostasis regulation and signal transduction of STAT1. RNF2 increased K33-linked polyubiquitination of the DNA-binding domain of STAT1 at position K379, resulting in the separation of STAT1 from DNA and consequently suppressing ISG transcription [11]. RNF220 mediated the K63-linked polyubiquitination of STAT1 at residue K110, which sustained the STAT1 activation through regulating the interaction between JAK1 and STAT1 [12]. Smurf1 promoted K48-linked polyubiquitination and proteasomal degradation of STAT1 to attenuated IFN-γ-mediated STAT1 activation and antiviral immune responses [13]. STAT1 harbors linear ubiquitination at K511 and K652 residues in intact cells, which inhibits STAT1 binding to the IFNAR2, thereby restricting STAT1 activation and resulting in IFN-I signaling homeostasis [14]. Viruses have hijacked the ubiquitin-proteasome system to evade innate immune responses and promote proper infection. Herpes simplex virus-2 (HSV-2) ICP22 acts as an E3 ubiquitin protein ligase to induce ubiquitination and degradation of STAT1 [15]. Simian virus 5 (SV5) and new castle disease virus (NDV) structural protein V induces the degradation of STAT1 [16,17]. The N protein of viral hemorrhagic septicemia virus (VHSV) interacts with STAT1 and promotes its K48-linked ubiquitination and proteasomal degradation [18]. Therefore, a comprehensive understanding of the molecular mechanism of STAT1 homeostasis regulation is crucial for antiviral drug screening and related disease prevention and treatment.

The exocyst complex is an evolutionarily conserved multisubunit protein complex, initially identified and characterized in budding yeast. The exocyst complex consists of eight subunits (Exoc1-8, originally named Sec3, Sec5, Sec6, Sec8, Sec10, Sec15, Exo70, and Exo84). Sec10 is the central subunit of the exocyst complex, primarily located in the cytoplasm [19]. Sec10 interacts with Sec8 through its C-terminus. Overexpression of Sec8 with N-terminal defects or Sec10 with C-terminal defects can disrupt the components of the endogenous extracellular secretory complex, thereby interfering with the growth of neurites [20]. Sec10 interacts with ciliary proteins polycytin-2, IFT88 and IFT20, and co-localizes with polycytin-2 in primary cilia, playing a role in cilia genesis and ciliary function [21]. Sec10 interacts with the translocation complex subunit Sec61b and participates in basolateral protein translation and translocation in the endoplasmic reticulum [22]. In addition, Sec10 interacts with the EGF receptor. Overexpression of Sec10 promotes ERK phosphorylation and activates the MAPK pathway, thereby facilitating EGFR endocytosis [23]. The individual components of the exocyst complex have been reported to be involved in viral infection. For example, Sec3 acts as a negative regulator of flavivirus infection by modulating viral RNA transcription and translation through sequestering elongation factor 1α [24]. Sec5 knockdown inhibits SARS-CoV-2 infection [25]. Sec6 can inhibit the singapore grouper iridovirus-induced apoptosis and promote the viral replication [26]. Exo70 promotes the virus egression/secretion from dengue virus-infected cells [27]. However, the role of Sec10 in the context of viral infections is not yet well understood.

In this study, we demonstrate that exocyst complex subunit Sec10 inhibits innate immunity and promotes viral replication by degrading STAT1. Mechanistic studies indicate that Sec10 promotes the interaction of STUB1 and STAT1, and catalyzes K6-linked polyubiquitination of Lys240 and Lys652 on STAT1 by STUB1, leading to the attenuation of type I IFN signaling and enhancement of viral replication.

## Results

### Sec10 inhibits the antiviral innate immune response

**Fig 1:**
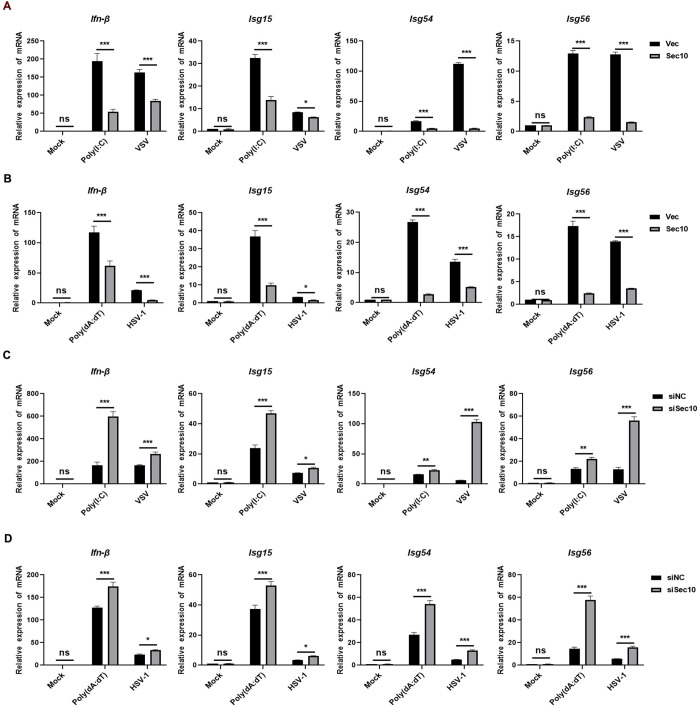
Sec10 suppresses the antiviral natural immune response. (A) HeLa cells were transfected with the Sec10-HA or empty vector (pLVX-IRES-puro) plasmid for 24 h and then treated with poly(I:C) (20 μg/mL) or infected with VSV (MOI = 1), and the transcription levels of *Ifn-β*, *Isg15*, *Isg54*, and *Isg56* were monitored by qRT-PCR. (B) qRT-PCR analysis of *Ifn-β*, *Isg15*, *Isg54*, and *Isg56* mRNA expression in HeLa cells transfected with the Sec10-HA or vector plasmid for 24 h and then treated with poly(dA:dT) (20 μg/mL) or infected with HSV-1 (MOI = 2). (C) HeLa cells were transfected with siNC or siSec10 and then treated with poly(I:C) (20 μg/mL) or infected with VSV (MOI = 1) for 12 h, and the transcription levels of **Ifn-*β, *Isg15**, *Isg54*, and *Isg56* were monitored by qRT-PCR. (D) HeLa cells were transfected with siNC or siSec10 followed by treated with poly(dA:dT) (20 μg/mL) or infected with HSV-1 (MOI = 2) for 12 h. qRT-PCR assays were performed to measure the mRNA levels of *Ifn-β*, *Isg15*, *Isg54*, and *Isg56.* Data are presented as the means ± SEM (*p < 0.05, **p < 0.01, ***p < 0.001, and ns indicates a nonsignificant difference) of three independent experiments.

To explore the function of Sec10 in the antiviral innate immune response, we transfected Sec10-HA and vector in HeLa cells. Quantitative real-time PCR (qRT-PCR) analysis showed that overexpression of Sec10 significantly attenuated the transcriptional levels of antiviral genes such as *Ifn-β*, *Isg15*, *Isg54*, and *Isg56* induced by transfection with poly(I:C) or by vesicular stomatitis virus (VSV) infection (Fig 1A). To determine whether Sec10 specifically mediates the innate immune response to DNA viruses, we infected Sec10-HA or vector HeLa cells with HSV-1 or stimulation with poly(dA:dT), and found that overexpression of Sec10 also downregulated the transcription of the *Ifn-β*, *Isg15*, *Isg54*, and *Isg56* genes (Fig 1B). Next, we knocked down Sec10 in HeLa cells using siRNA (S1A Fig), qRT-PCR results indicated that Sec10 knockdown significantly enhanced the mRNA levels of the *Ifn-β*, *Isg15*, *Isg54*, and *Isg56* genes induced by transfection with poly(I:C) or by VSV infection (Figs 1C and S1B). Consistently, we observed that Sec10 knockdown enhanced the mRNA level of *Ifn-β*, *Isg15*, *Isg54*, and *Isg56* upon stimulation with poly(dA:dT) or infection with HSV-1 (Fig 1D). To further investigate the role of Sec10 in antiviral immune responses in vivo, we generated Sec10^*fl/+*^ mice by CRISPR/Cas9-mediated genome editing. Sec10^*fl/+*^ mice was hybridized with *Lyz*2-Cre transgenic mice, in which exon 7–10 of Sec10 was specifically deleted by Cre recombinase expression on bone marrow cell-specific promoter Lyz. We analyzed the expression of IFNβ and ISGs in bone marrow-derived macro phages (BMDMs) and peritoneal macrophages (PMs) from *Lyz*2-Cre;Sec10^*fl/fl*^ and Sec10^*fl/fl*^ mice, and found that the mRNA levels of *Ifn-β*, *Isg15*, *Isg54*, *Isg56* and the production of IFNβ were also increased in Sec10-deficient BMDMs and PMs infected with VSV or transfected with poly(I:C) and poly(dA:dT) (S1C–S1F Fig). Moreover, we then examined the expression of proinflammatory cytokines in Sec10-deficient BMDMs and PMs, and found the mRNA levels of IL-6 and TNF-α were also increased (S1G and S1H Fig). Taken together, these data suggest that Sec10 suppresses the RNA and DNA viruses triggered-antiviral innate immune response.

### Sec10 abrogates the JAK-STAT pathway via inhibits STAT1

**Fig 2:**
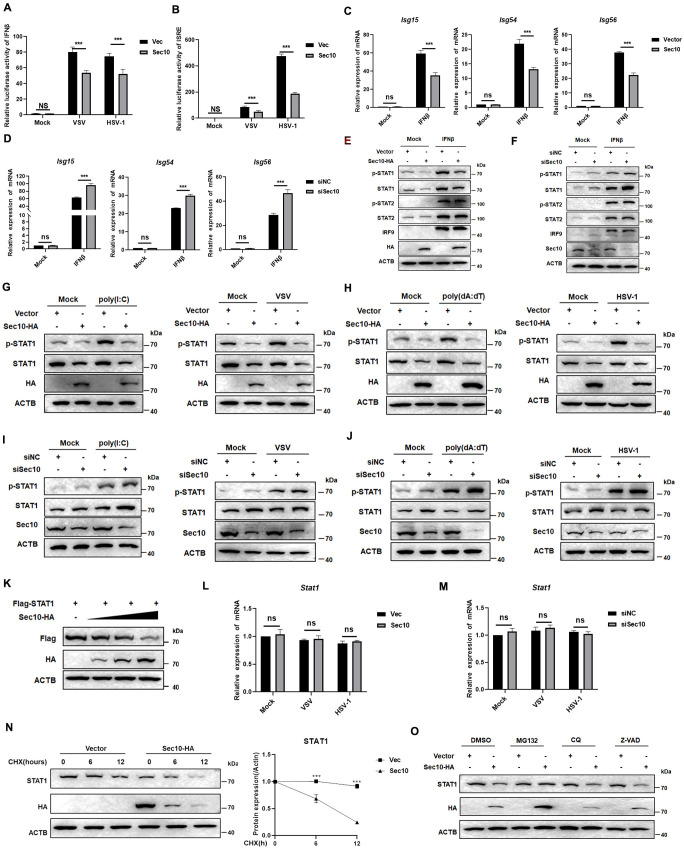
Sec10 blocks the JAK-STAT pathway by downregulating the expression of STAT1. (A and B) Luciferase reporter assays analyzing IFN-β or IFN-stimulated response element (ISRE) promoter activity of HEK293T cells transfected with Sec10-HA or empty vector for 24 h, followed by treatment with or without VSV (MOI = 1) or HSV-1 (MOI = 2) for 12 h, respectively. (C) HeLa cells were transfected with control vectors or Sec10 expression plasmids were stimulated with IFNβ (20 ng/mL) for 12 h. Then, the induction of *Isg15*, *Isg54*, and *Isg56* mRNAs was measured by qRT-PCR. (D) HeLa cells were transfected with siNC or siSec10 for 24 h and then treated with IFNβ, and the transcription levels of *Isg15*, *Isg54*, and *Isg56* were monitored by qRT-PCR. (E) HeLa cells were transfected with the Sec10-HA or vector plasmid and then treated with IFNβ. The cell lysates were subjected to immunoblotting analysis with the indicated antibody. (F) HeLa cells were transfected with siNC or siSec10 followed by treated with IFNβ. The cell lysates were subjected to immunoblotting analysis with the indicated antibody. (G and H) HeLa cells were transfected with the Sec10-HA or vector plasmid for 24 h and then treated with poly(I:C) (20 μg/mL) and poly(dA:dT) (20 μg/mL) or infected with VSV (MOI = 1) and HSV-1 (MOI = 2). The cell lysates were subjected to immunoblotting analysis with the indicated antibody. (I and J) HeLa cells were transfected with siNC or siSec10 and then treated with poly(I:C) (20 μg/mL) and poly(dA:dT) (20 μg/mL) or infected with VSV (MOI = 1) and HSV-1 (MOI = 2). The cell lysates were subjected to immunoblotting analysis with the indicated antibody. (K) Immunoblotting analysis of the expression of STAT1 in HEK293T cells transfected with the Flag-Sec10 plasmid (0, 400, 800 and 1600 ng) and then infected with VSV. (L) HeLa cells were transfected with empty vector or Sec10-HA, followed by VSV (MOI = 1) or HSV-1 (MOI = 2) infection for 12 h, and the levels of STAT1 expression was monitored by qRT-PCR. (M) HeLa cells were transfected with siNC or siSec10, followed by VSV (MOI = 1) or HSV-1 (MOI = 2) infection for 12 h, and the levels of STAT1 expression was monitored by qRT-PCR. (N) Immunoblot analysis of protein extracts of HeLa cells transfected with plasmids encoding empty vector or Sec10-HA, subjected to VSV infection, followed by CHX treatment for the indicated time points. (O) HeLa cells transfected with the Sec10-HA and empty vector, followed by VSV infection for 12 h. Protein lysates of the cells treated with MG132 (5 μM), CQ (20 μM), or Z-VAD (10 nM) for 12 h. The cell lysates were then analyzed by western blotting. Data are presented as the means ± SEM (*p < 0.05, **p < 0.01, ***p < 0.001, and ns indicates a nonsignificant difference) of three independent experiments.

Given that, we next sought to explored the mechanism by which Sec10 inhibits antiviral immune responses. By overexpressing Sec10 in HEK293T cells, we found that Sec10 markedly suppressed activation of IFN-β-and IFN-stimulated response element (ISRE)–responsive reporters induced by VSV or HSV-1 (Fig 2A and 2B). Moreover, we found that Sec10 inhibits the ISGs expression induced by IFNβ. We treated Sec10-HA HeLa cells or control cells with IFNβ for 12 h, and then detected the transcriptional levels of ISGs genes such as *Isg15*, *Isg54*, and *Isg56*. The data showed that overexpression of Sec10 significantly attenuated the transcriptional levels of *Isg15, Isg54, and Isg56* induced by treatment with IFNβ (Fig 2C). Sec10 knockdown enhanced the mRNA level of *Isg15, Isg54, and Isg56* upon stimulation with IFNβ (Fig 2D). Moreover, we observed that IFNβ-induced the mRNA level of *Isg15, Isg54, and Isg56* were enhanced in BMDMs and PMs from *Lyz*2-Cre;Sec10^*fl/fl*^ mice compared with Sec10^*fl/fl*^ mice (S2A Fig), indicating that Sec10 can block the downstream signaling transduction of IFN triggered by the viral infection.

JAK-STAT pathway is downstream of the IFN signaling pathway co-activated by RLRs and cGAS/STING, major PRRs that recognize viral infection [28]. Because Sec10 can simultaneously block IFN response triggered by RNA and DNA virus infection and inhibit the expression of ISGs. Therefore, we speculate that Sec10 may be involved in the regulation of the JAK-STAT signaling pathway. To test this hypothesis, we next examined the effect of Sec10 on the expression of key proteins in the JAK-STAT signaling pathway and found that Sec10 reduced the levels of phosphorylated STAT1 and STAT1 but did not affect the expression of phosphorylated STAT2, STAT2, and IRF9 (Fig 2E–2J). In addition, we found that the expression of p-STAT1 and STAT1 were enhanced in Sec10-deficient BMDMs and PMs than that in wild-type macrophages upon infection with VSV and HSV-1 or stimulation with poly(I:C) and poly(dA:dT) (S2B and S2C Fig). HEK293T cells were transfected with the STAT1 and different doses of Sec10 plasmid, after which the cells were harvested and subjected to immunoblotting analysis, and found that Sec10 downregulated the expression of STAT1 in a dose-dependent manner (Fig 2K). These data indicated that Sec10 downregulated JAK-STAT pathway by inhibiting the expression of STAT1.

Next, we explored the mechanism by which Sec10 down-regulated STAT1 expression. First, we examined whether Sec10 affected the transcriptional expression of STAT1, and found that overexpression of Sec10 did not affect the mRNA expression of STAT1 induced by viral infection (Fig 2L and 2M). To verify whether the accumulation of STAT1 is affected by Sec10, we treated Sec10-HA HeLa cells and control cells with cycloheximide (CHX), and detected STAT1 protein levels for the indicated times. The results showed that overexpression of Sec10 decreased the half-life of endogenous STAT1 protein after CHX treatment (Fig 2N). Therefore, we then explored whether Sec10 regulates STAT1 proteostasis via the Ub-proteasome system, autophagosomal pathway or caspase pathway, we expressed Sec10 in HeLa cells and then treated the cells with the protease inhibitor MG132, the autophagy inhibitor Chloroquine (CQ), and pan-caspase inhibitor Z-VAD-FMK. Sec10-mediated degradation of the STAT1 protein was blocked by MG132 but not the CQ and Z-VAD-FMK (Fig 2O). Collectively, these data indicate that Sec10 promotes degradation of STAT1 protein via the proteasome pathway.

### Sec10 promotes the Lys6-linked polyubiquitination of STAT1 at Lys240 and Lys652

**Fig 3:**
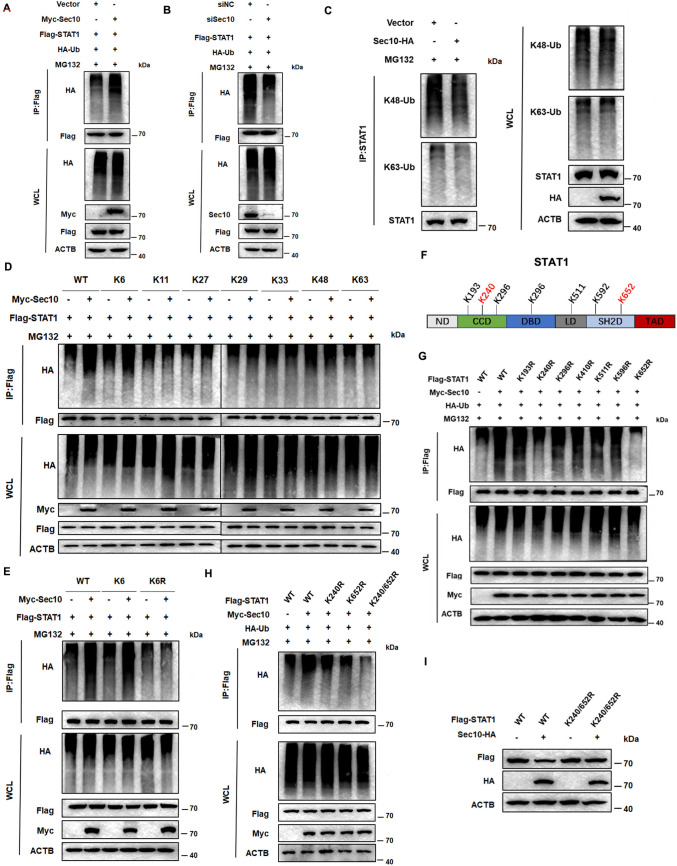
Sec10 catalyzes Lys6-linked polyubiquitination of STAT1 on Lys240 and 652. (A) HeLa cells were transiently transfected with Myc-Sec10 or vector together with Flag-tagged STAT1 and HA-Ub as indicated for 48 h. Then, polyubiquitination of STAT1 was examined by Western blotting after IP with anti-Flag Sepharose beads. (B) HeLa cells were transfected with siNC or siSec10 together with Flag-tagged STAT1 and HA-Ub as indicated for 48 h. Then, polyubiquitination of STAT1 was examined by Western blotting after IP with anti-Flag Sepharose beads. (C) Immunoprecipitation analysis lysates from HEK293T cells transiently cotransfected with K48-Ub, K63-Ub, Sec10-HA, or vector. (D) IP analysis lysates from HEK293T cells transiently cotransfected with HA-Ub (WT and its mutants), Myc-Sec10, and Flag-STAT1. (E) Lysates from HEK293T cells transiently cotransfected with Flag-STAT1, Myc-Sec10, and K6-Ub or K6R-Ub, were subjected to immunoprecipitation with Flag antibody. (F-H) IP analysis lysates from HEK293T cells transiently cotransfected with HA-Ub, Myc-Sec10, along with Flag-STAT1 (WT and its point mutants). (I) Immunoblot analysis of extracts from HEK293T cells transfected with Flag-STAT1 or Flag-STAT1 K240/652R mutant, together with Sec10-HA plasmid.

The lysine residues on the substrate undergoing polyubiquitination are crucial for the ubiquitin-proteasome degradation pathway [29]. First, we investigated the effects of Sec10 on STAT1 ubiquitylation. Polyubiquitination of STAT1 was substantially increased in the presence of Sec10 expression plasmid in HeLa cells (Fig 3A). Knockdown of Sec10 leads to a reduction in the ubiquitination modification of STAT1 (Fig 3B). Lys48 (K48) linked polyubiquitin chains are well established as the canonical signal for proteasomal degradation, K63-polyubiquitin conjugates are involved in non-proteasomal pathways [30]. Next, we investigate whether Sec10 directly catalyzes the K48-linked polyubiquitination modification of STAT1, the K63 ubiquitination as a control, and found that Sec10 could not catalyze the polyubiquitination of STAT1 at K48 and K63 (Fig 3C). In order to decipher the ubiquitin chain linkage catalyzed by Sec10 on STAT1, we expressed five additional ubiquitin mutant forms. Our findings revealed that the polyubiquitination of STAT1 could be detected in the presence of K6 ubiquitin variant, whereas it was undetectable in the other mutant forms (Fig 3D). Furthermore, upon expressing the K6R variant (lysine residue is replaced with arginine) in HEK293T cells, we observed that Sec10 no longer facilitated the ubiquitination modification of STAT1 (Figs 3E and S3A). Next, we aim to identify the lysine residues in STAT1 that are susceptible to ubiquitination. Using the UbPred program, we predict that STAT1 harbors seven potential ubiquitination sites (Fig 3F). Then, we replaced each of the seven STAT1 lysine (K) residues individually with arginine (R) to create K193R, K240R, K296R, K410R, K511R, K596R, and K652R mutants of STAT1, respectively. IP assay revealed that only K240R, and K652R partly blocked the ubiquitination of STAT1 mediated by Sec10 (Fig 3G). Sec10-mediated ubiquitination and protein degradation of STAT1 were completely abolished in STAT1 K240/652R double-site mutant (Figs 3H,3I,S3B, and S3C). Thus, these results suggest that Sec10 catalyzes Lys6-linked polyubiquitination of STAT1 on Lys240 and Lys652.

### STUB1 is necessary for Sec10-mediated degradation of STAT1 and inhibits the IFN-I responses

**Fig 4:**
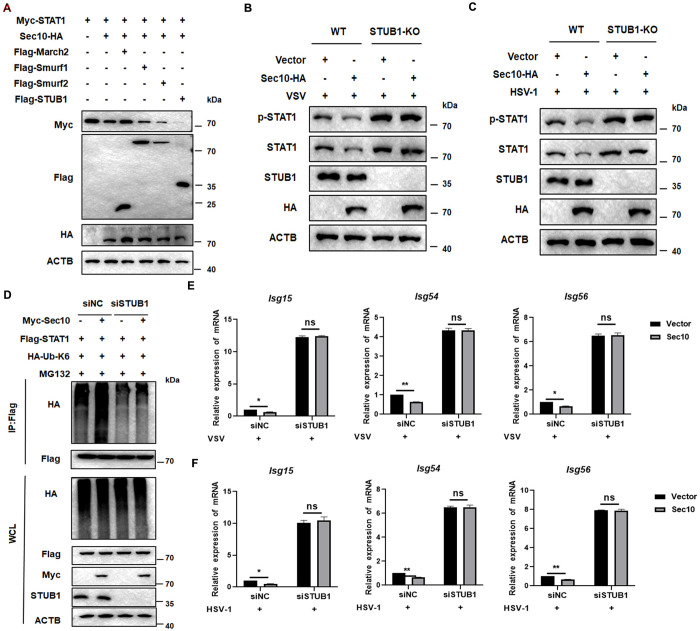
STUB1 is crucial for Sec10-mediated the degradation of STAT1 and ISGs production. (A) Immunoblotting analysis the expression of STAT1 using lysates from HEK293T cells were transfected with vectors encoding Sec10-HA, and indicated E3 ubiquitin ligase. (B and C) Immunoblot analysis of extracts of STUB1-KO HeLa cells transfected with Sec10-HA or vector followed by VSV or HSV-1 infection for indicated time points. (D) HEK293T cells that expressed siNC or siSTUB1 were transiently transfected with Myc-Sec10 or vector together with Flag-tagged STAT1 and HA-Ub-K6 as indicated for 48 h. Then, polyubiquitination of STAT1 was examined by Western blotting after IP with anti-Flag Sepharose beads. (E and F) HeLa cells that stably expressing vector and Sec10-HA were transfected with siNC or siSTUB1, followed by VSV (MOI = 1) or HSV-1 (MOI = 2) infection for 12 h, and the *Isg15*, *Isg54*, and *Isg56* mRNA expression was monitored by qRT-PCR. Data are presented as the means ± SEM of three independent experiments.

The observed findings implicate that Sec10 catalyzes Lys6-linked polyubiquitination and degradation of STAT1. Given Sec10’s inherent lack of E3 ubiquitin ligase activity, it is inferred that E3 ubiquitin ligases might play a role in the Sec10-mediated K6-linked polyubiquitination and proteasomal degradation of STAT1. To test this, we used the UbiBrowser prediction software to identify potential E3 ubiquitin ligases that might interact with STAT1. Subsequently, the selected candidate E3 ligases and Sec10 were co-transfected into HEK293T cells and STAT1 expression was detected. We found that STUB1 promoted the degradation of STAT1 by Sec10, while other E3 ligases had no significant effect (Fig 4A). To elucidate the role of STUB1 in Sec10 ubiquitination modification and degradation of STAT1, we silenced STUB1 in HeLa cells that stably expressing Sec10. Immunoblot analysis demonstrated that the knockout of STUB1 blocked the Sec10-mediated degradation of STAT1 and inhibition of p-STAT1 upon infection with VSV or HSV-1 (Fig 4B and 4C). Consistent with these findings, Sec10 could not catalyze the K6-linked polyubiquitination of STAT1 in HEK293T cells that knocked down STUB1 (Fig 4D). Then, whether STUB1 intervene in the regulation of Sec10-mediated antiviral IFN-JAK-STAT signaling response by degrading STAT1. As shown in Fig 4E and 4F, knockdown of STUB1 expression inhibited the inhibitory effect of Sec10 on the transcriptional levels of *Isg15*, *Isg54*, and *Isg56* triggered by infection with VSV or HSV-1. In brief, these results indicate that Sec10 inhibits the antiviral IFN-JAK-STAT signaling response by regulating STAT1 expression in an STUB1-dependent manner.

### STUB1 interacts with the TAD domain of STAT1 via its TPR domain

**Fig 5:**
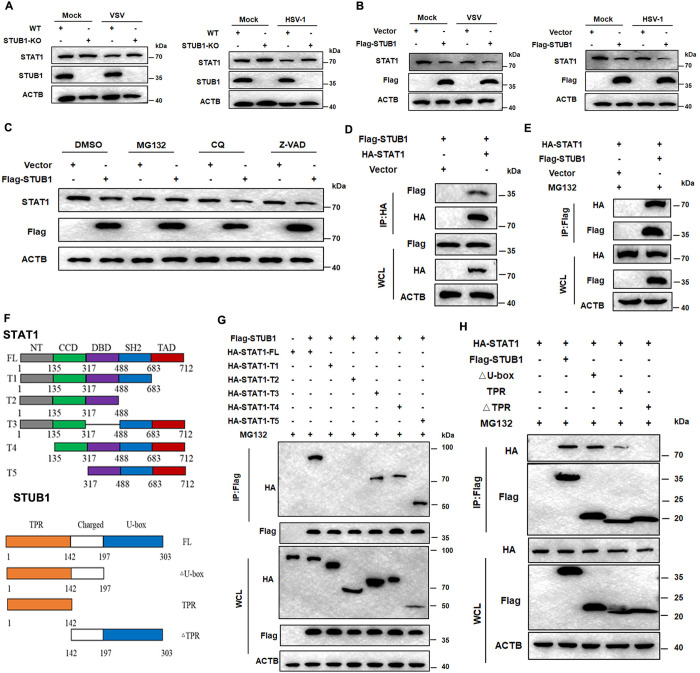
STUB1 interacts with STAT1. (A) Immunoblotting analysis of the expression of STAT1 in STUB1-KO or WT HeLa cells that infected with VSV (MOI = 1) or HSV-1 (MOI = 2). (B) Immunoblotting analysis of the expression of STAT1 in HeLa cells transfected with the Flag-STUB1 or vector plasmid and then infected with VSV (MOI = 1) or HSV-1 (MOI = 2) for 12 h. (C) HeLa cells transfected with the Flag-STUB1 and empty vector, followed by VSV infection for 12 h. Protein lysates of the cells treated with MG132 (5 μM), CQ (20 μM), or Z-VAD (10 nM) for 12 h. The cell lysates were then analyzed by western blotting. (D and E) Immunoblotting analysis was performed to detect the interaction between STUB1 and STAT1. The cell lysates were immunoprecipitated with anti-HA or anti-Flag antibody and immunoblotted with anti-Flag or anti-HA antibody, respectively. (F) A schematic diagram of STAT1 and STUB1 functional domains. (G) Co-immunoprecipitation and immunoblot analysis of HEK293T cells cotransfected with Flag-STUB1 with HA-STAT1 or HA-STAT1 truncated vectors. (H) Co-immunoprecipitation and immunoblot analysis of HEK293T cells cotransfected with HA-STAT1 with Flag-STUB1 or Flag-STUB1 truncated vectors.

We next investigated whether STUB1 could promote the protein degradation of STAT1. We generated STUB1-knockout (KO) HeLa cells using a CRISPR-Cas9-based approach, and examined the levels of endogenous STAT1 protein in STUB1-KO HeLa cells. STAT1 protein was significantly increased in STUB1-KO HeLa cells, similar to that observed in siSec10 HeLa cells (Fig 5A). STUB1 overexpression inhibited STAT1 expression in HeLa cells (Fig 5B). Moreover, STUB1-mediated degradation of the STAT1 protein was blocked by MG132 (Fig 5C). Next, we examined whether STUB1 interacted with STAT1. We cotransfected HEK293T cells with Flag-tagged STUB1 and HA-tagged STAT1 plasmids, and subjected them to immunoprecipitation and immunoblot analysis with anti-HA or anti-Flag antibodies. The data showed that STUB1 could be coimmunoprecipitated (Co-IP) with STAT1 (Fig 5D and 5E). Then, we aimed to determine which structural domain is responsible for the interaction between STUB1 and STAT1. Co-IP assays were performed using the STAT1 and STUB1 truncation mutants (Fig 5F). We found that the truncated STAT1 mutant lacking the TAD domain abolished the capacity for STUB1 binding, indicating that the TAD domain mediates interaction with STUB1 (Fig 5G). The truncated STUB1 mutant lacking the TPR domain significantly reduced its interaction with STAT1, suggesting that the TPR domain is essential for the interaction between STAT1 and STUB1 (Fig 5H). Taken together, these results demonstrate that STUB1 binds to the TAD domain of STAT1 through its TPR domain and degrades STAT1.

### Sec10 is essential for interaction between STAT1 and STUB1

**Fig 6:**
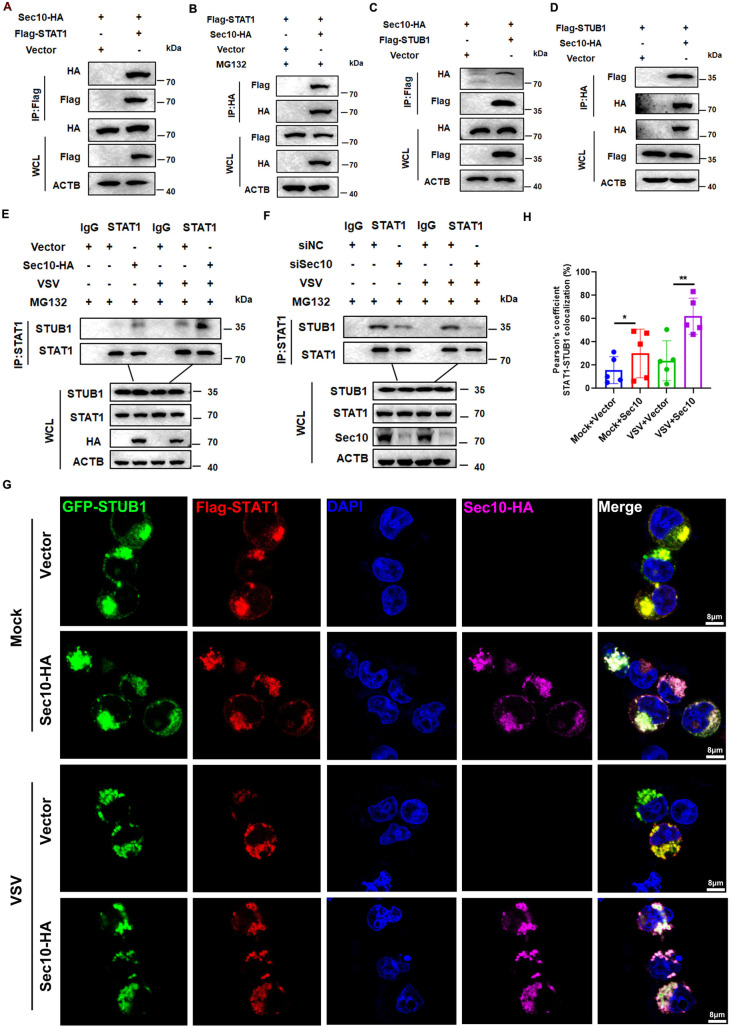
Sec10 facilitates the interaction between STUB1 and STAT1. (A and B) Co-immunoprecipitation and immunoblot analysis of HEK293T cell cotransfected with Sec10-HA and Flag-STAT1. (C and D) Co-immunoprecipitation and immunoblot analysis of HEK293T cell cotransfected with Sec10-HA and Flag-STUB1. (E) Lysates of HeLa cells stably expressed Sec10-HA or empty vector and infected with VSV for 12 h and treatment with MG132 for 12 h. The lysates were subjected to IP with anti-STAT1 and immunoblot analysis with indicated antibodies (shown on the left). (F) Lysates of HeLa cells transfected with siSec10 or siNC and infected with VSV for 12 h and treatment with MG132 for 12 h. The lysates were subjected to IP with anti-STAT1 and immunoblot analysis with indicated antibodies (shown on the left). (G) HeLa cells were transfected with Flag-STAT1, GFP-STUB1, and Sec10-HA plasmid or empty vector for 36 h. The cells were then immunostained for Sec10 (pink), STAT1 (red) and fluorescence signals were visualized by confocal immunofluorescence microscopy. Scale bars: 8μm. The fluorescence intensity profiles of Sec10 (pink), STAT1 (red), and STUB1 (green) were measured along the line drawn by ImageJ. (H) Quantitative analysis of the colocalization in (G). Data are presented as the means ± SEM of three independent experiments.

To ascertain the function of Sec10 in the binding of STAT1 with STUB1, we overexpressed Sec10 and STAT1 in HEK293T cells and conducted Co-IP assays. As shown in Fig 6A and 6B, overexpressed HA-tagged Sec10 was associated with Flag-tagged STAT1. STUB1 was shown to interact with Sec10 by reciprocal Co-IP experiments (Fig 6C and 6D). In addition, endogenous Co-IP also confirmed the interaction between Sec10, STUB1, and STAT1 (S4A and S4B Fig). Next, we examined the association between STAT1 and STUB1 in the presence or absence of Sec10. Co-IP analysis showed that overexpression of Sec10 could significantly enhance the endogenous association between STAT1 and STUB1, and silencing Sec10 leads to a decrease in endogenous interactions between STAT1 and STUB1 (Fig 6E and 6F). Moreover, confocal microscopy analysis revealed that overexpression of Sec10 significantly increased the extent of colocalization between STAT1 and STUB1 (Fig 6G and 6H). These results indicate that Sec10 plays an essential role in mediating the interaction between STAT1 and STUB1.

### Sec10 facilitates viral replication via Sec10-STUB1-STAT1 axis

**Fig 7:**
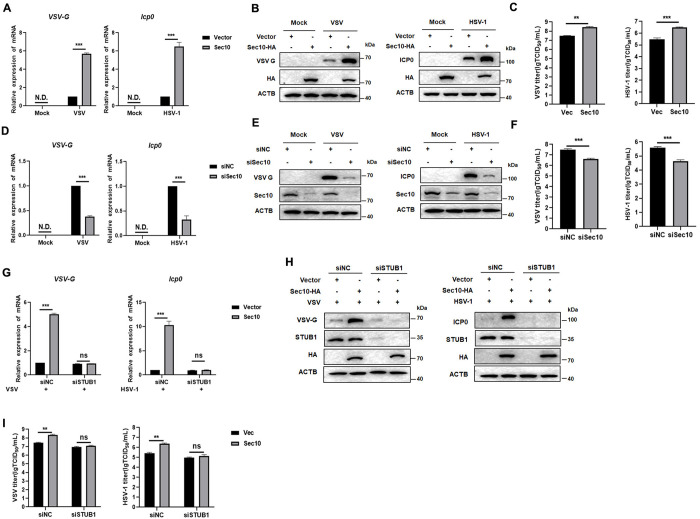
Sec10 promotes viral replication through Sec10-STUB1-STAT1 pathway. (A-C) HeLa cells were transfected with the Sec10-HA or empty vector plasmid for 24 h and then infected with VSV (MOI = 1) or HSV-1 (MOI = 2), and the transcription levels of *VSV-G* and *Icp0* were monitored by qRT-PCR (A), and the cell lysates were subjected to immunoblotting analysis with the indicated antibodies (B), and the viral titer was determined by TCID_50_ assay (C). (D-F) After transfection with siNC or siSec10, HeLa cells were infected with VSV (MOI = 1) or HSV-1 (MOI = 2) for 12 h, and the transcription levels of *VSV-G* and *Icp0* were monitored by qRT-PCR (D), and the cell lysates were subjected to immunoblotting analysis with the indicated antibodies (E), and the viral titer was determined by TCID_50_ assay (F). (G-I) HeLa cells that stably expressing vector and Sec10-HA were transfected with siNC or siSTUB1, followed by VSV (MOI = 1) or HSV-1 (MOI = 2) infection for 12 h, and the transcription levels of *VSV-G* and *Icp0* were monitored by qRT-PCR (G), and the cell lysates were subjected to immunoblotting analysis with the indicated antibodies (H), and the viral titer was determined by TCID_50_ assay (I). Data are presented as the means ± SEM of three independent experiments.

Next, we examined the effects of Sec10 on viral propagation. HeLa cells transfected with the Sec10-HA or vector plasmid were infected with VSV or HSV-1 and harvested at the indicated time points. The cell lysates were analyzed by western blotting and qRT-PCR. The protein and mRNA levels of the VSV-G and HSV-1 ICP0 were upregulated in Sec10-HA-transfected cells compared to control cells (Fig 7A and 7B). The TCID_50_ assay showed that the viral titer in Sec10-HA cells was higher than control cells (Fig 7C). Consistent with these observations, we silenced the expression of Sec10 in viral-infected cells and found that the protein and mRNA levels of the VSV-G and HSV-1 ICP0 protein were significantly reduced (Fig 7D and 7E). TCID_50_ assay shows that inhibition the expression of Sec10 reduced the viral replication (Fig 7F). In summary, Sec10 disrupts innate immunity and promotes viral replication. We hypothesized that the roles of Sec10 in innate antiviral response and viral replication might rely on its promotion of STUB1’s ubiquitination modification and degradation of STAT1. So, we silenced STUB1 in HeLa cells that stably expressing Sec10, and found that overexpression of Sec10 no longer had the ability to promote virus replication in siSTUB1 cells (Fig 7G–7I). These results suggested that Sec10 promotes viral replication through Sec10-STUB1-STAT1 pathway.

### Sec10 deficiency leads to decreased susceptibility to lethal VSV infection

**Fig 8:**
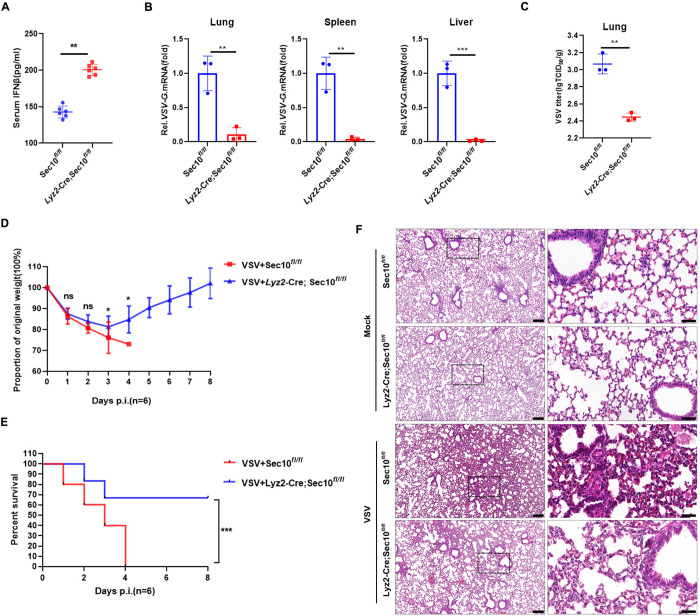
Sec10 negatively regulates antiviral innate immunity in mice. (A) ELISA for IFNβ in serum of Sec10^*fl/fl*^ and *Lyz*2-Cre;Sec10^*fl/fl*^ mice infected with VSV(1 × 10^8^ PFU per mouse) via intraperitoneal injection for 18 h. (B) Sec10^*fl/fl*^ and *Lyz*2-Cre;Sec10^*fl/fl*^ mice were injected intraperitoneally with VSV(1 × 10^8^ PFU per mouse) for 18 h. The relative induction of VSV-G mRNA in lungs, Livers, and spleens from Sec10^*fl/fl*^ and *Lyz*2-Cre;Sec10^*fl/fl*^ mice was measured by qRT-PCR (n = 3 per group). (C) TCID_50_ assays for viral titers in lungs of Sec10^*fl/fl*^ and *Lyz*2-Cre;Sec10^*fl/fl*^ mice injected intraperitoneally with VSV (1 × 10^8^ PFU per mouse) for 3 days (n = 3 per group). (D and E) Age- and sex-matched Sec10^*fl/fl*^ and *Lyz*2-Cre;Sec10^*fl/fl*^ mice (7 weeks of age) were intraperitoneal infected with VSV(1 × 10^8^ PFU per mouse) or PBS, and changes in the survival rate and body weights of the mice were recorded. (F) Hematoxylin and eosin (HE) staining of lung sections isolated from Sec10^*fl/fl*^ and *Lyz*2-Cre;Sec10^*fl/fl*^ mice infected with VSV by intraperitoneal injection for 48 h. Scale bar, 200 μm. Data are presented as the means ± SEM of three independent experiments.

To investigate the physiological role of Sec10 in antiviral responses in vivo, *Lyz*2-Cre;Sec10^*fl/fl*^ and Sec10^*fl/fl*^ mice were intraperitoneally injected with VSV. Our results showed that the VSV-induced IFNβ secretion was significantly increased in the serum from *Lyz*2-Cre;Sec10^*fl/fl*^ mice compared to that from Sec10^*fl/fl*^ mice (Fig 8A). In the spleen, liver, and lung of *Lyz*2-Cre;Sec10^*fl/fl*^ mice infected with VSV, the expression of VSV-G was lower than that of Sec10^*fl/fl*^ mice (Fig 8B). Consistent with these findings, the VSV titers in the lung of *Lyz*2-Cre;Sec10^*fl/fl*^ mice were lower than in the Sec10^*fl/fl*^ mice at day 2 after VSV infection (Fig 8C). In addition, the *Lyz*2-Cre;Sec10^*fl/fl*^ mice presented less susceptibility and slighter weight loss to VSV-induced lethality than the Sec10^*fl/fl*^ mice (Fig 8D and 8E). Hematoxylin and eosin staining revealed that more severe inflammation and immune cell infiltration in the lungs of Sec10^*fl/fl*^ mice compared with *Lyz*2-Cre;Sec10^*fl/fl*^ mice after VSV infection (Fig 8F). Collectively, these data suggest that the Sec10 is a negative regulator of IFN-JAK-STAT signaling in response to VSV infection.

## Discussion

Upon viral infection, the activation of host PRRs triggers the production of IFNs, which in turn activates the JAK-STAT pathway, leading to a potent immune response against the virus [31]. STAT1, by transmitting IFN signals, facilitates the transcription of genes with critical antiviral properties, playing a central role in the immune reaction to viral infection [4]. Here, we demonstrate that the exocyst complex subunit Sec10 acts as a specific negative regulator for STAT1-mediated innate antiviral immune responses, blocking IFN-I signal transduction and promoting viral replication.

Sec10 is a core component of the highly-conserved octameric exocyst protein complex, which mediates the targeting and docking of intracellular vesicles [32,33]. The exocyst complex subunit Sec5 has been shown to significantly impact the antiviral innate immunity response [26,34,35], but the exact functions of the other subunits in the host’s defense against viral infections are not yet well understood. In this study, we evaluated the function of Sec10 in antiviral innate immunity by enhancing or inhibiting Sec10 at the cellular level. Our findings indicate that overexpression of Sec10 inhibited the transcriptional levels of IFN-β and ISGs induced VSV or HSV-1 infection. Conversely, knockdown of Sec10 increased the levels of IFN-β and ISGs induced by transfection with RNA or DNA virus analogs or by viral infection. This suggests that Sec10 can inhibit both RNA or DNA virus-induced IFN-I response. We further generated Sec10 knockout mice and tested the impact of Sec10 deletion on mouse resistance to viral infection in vivo. Given that general murine knockout of exocyst genes has proved to be early embryonic lethal [36]. Thus, we created myeloid-specific Sec10 knockout mice, and found that IFN-I and ISGs levels were higher in *Lyz*2-Cre;Sec10^*fl/fl*^ PMs or BMDMs compared with Sec10^*fl/fl*^ PMs upon VSV infection. These results suggest that Sec10 can inhibit the antiviral innate immune response in cells and mice. However, the mechanisms by which Sec10 regulates IFN-I signaling responses induced by RNA and DNA viruses remain to be further explored.

The JAK/STAT pathway plays a pivotal role in immune regulation, being activated by interferons (IFNs) induced by both RNA and DNA viruses [37]. STAT1 plays an important role in IFN-stimulated cellular signal transduction. The selective gene deletion of STAT1 in mice or the presence of loss-of-function mutations of STAT1 in humans both cause rapid death from severe infections [4]. Our research indicates that Sec10 is capable of suppressing the expression of STAT1 in cells and mice. Viruses can evade the host’s antiviral innate immune response by degrading STAT1 protein, inhibiting its activation, promoting the dephosphorylation, or inducing the expression of its β isoform [38,39]. We discovered that Sec10 degrades STAT1 through the ubiquitin-proteasome pathway to block the antiviral innate immune response. The ubiquitination modification of substrates is a critical step in the ubiquitin-proteasome pathway. STAT1 can undergo K33-, K48-, and K63-linked polyubiquitination modification, as previously reported [11–13]. However, K6, K11, K27, and K29 types of STAT1 polyubiquitinations have not been reported. Our study identifies a previously unidentified type of STAT1 polyubiquitination, K6-linked polyubiquitination. We found that Sec10 promotes the K6-linked polyubiquitination of STAT1 at K240 and K652. It has been reported that STAT1 K652 can undergo linear ubiquitination modification, and linear ubiquitination of STAT1 inhibits its interaction with the membrane receptor IFNAR2, which finally blocks IFN-I induced STAT1 activation [14]. However, the stability of STAT1 protein was not affected. In our study, the polyubiquitination of K6-linked STAT1 K652 resulted in the recognition and degradation of STAT1 by the proteasome, and the total protein amount of STAT1 was reduced.

Sec10 lacks enzymatic activity and cannot catalyze the ubiquitination of STAT1. Therefore, we further investigated the E3 ubiquitin ligase involved in the degradation of STAT1 by Sec10. We found that E3 ubiquitin ligase STUB1 enhanced Sec10-mediated degradation of STAT1, and found that Sec10, STUB1 and STAT1 interact with each other. Sec10 facilitates the association between STUB1 and STAT1. STUB1 also known as CHIP, is a U-box type chaperone associated E3 ligase [40]. STUB1 has been reported to mediate the ubiquitination and degradation of key immune system factors [41–43]. Here, we reveal a new function of STUB1 that degrades STAT1 via the proteasome pathway. The primary organization of STUB1’s structure comprises three structured motifs: an N-terminus tetrapeptide repeats sequence (TPR) domain, a central coil domain, and a C-terminus U-box domain [44]. STAT1 protein structure includes six regions: the N-terminal domain (ND), coiled-coil domain (CCD), DNA binding domain (DBD), linker domain (LD), Src homology-2 domain (SH2D), and TAD [45]. We found that STUB1 binds to the TAD domain of STAT1 through its TPR domain and degrades STAT1. Importantly, STUB1 is necessary for Sec10-mediated K6-linked polyubiquitination and degradation of STAT1.

Furthermore, we have investigated the role of Sec10 in viral replication and found that Sec10 enhances the replication of VSV and HSV-1. Silencing of STUB1 leads to the disappearance of the enhancement effect of Sec10 on viral replication, indicating that Sec10 inhibits antiviral innate immune response and promotes viral replication through Sec10-STUB1-STAT1 axis. This view has also been supported by in vivo experiments. Sec10 deficiency leads to accumulation of STAT1 protein in mice. Sec10-deficient mice produced more IFN-I in response to virus infection and exhibited enhanced innate immune responses, and reduced viral load and morbidity in vivo. In brief, these findings reveal a previously unrecognized role of Sec10 in regulating antiviral innate immune responses and viral replication. In addition, although this study focuses on STAT1, Sec10 could target other signaling proteins of the IFN signaling pathway, which will be investigated and presented in a separate study.

**Fig 9:**
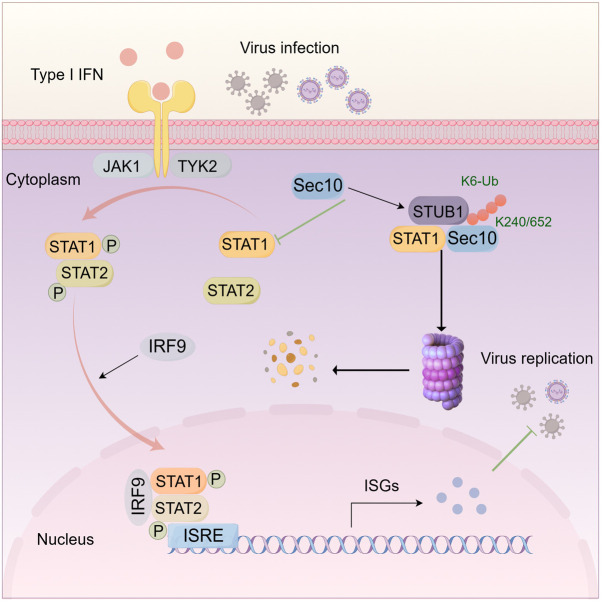
Schematic diagram of Sec10 blocking innate immunity and promoting viral replication by degrading STAT1. The schematic diagram was created by the authors using Figdraw, with permission for image download and publication granted by Figdraw.

In this study, we report the mechanism by which the Sec10 abrogates antiviral innate immunity and facilitates viral replication (Fig 9). Based on our findings, Sec10 interacts with STUB1 and STAT1, and facilitates STUB1-mediated proteasomal degradation of STAT1, subsequently leading to the blockade of STAT1-mediated antiviral signals. The insights from these studies will contribute to a deeper understanding of novel role of Sec10 in antiviral innate immunity, as well as the development of Sec10 as a potential target for clinical antiviral drugs.

## Materials and methods

### Ethical approval

This study was performed in strict accordance with the recommendations in the Guide for the Care and Use of Laboratory Animals of the Institute of Microbiology, Chinese Academy of Sciences (IMCAS) Ethics Committee, and all experiments conform to the relevant regulatory standards. The experiments and protocol were approved by the Committee on the Ethics of Animal Experiments of IMCAS. All animal experiments were conducted under isoflurane anesthesia to minimize animal suffering. Studies with HSV-1 were conducted under biosafety level 2 (BSL2) and animal BSL3 (A-BSL3) containment.

### Cells and viruses

HEK293T (ATCC CRL-11268) and HeLa (ATCC CCL-2) cells were cultured in Dulbecco’s modified Eagle’s medium (DMEM; BI) supplemented with 10% fetal bovine serum (FBS; HyClone), penicillin and streptomycin (100 U/mL; New Cell & Molecular Biotech Co., Ltd). Cells were maintained at 37˚C in a 5% CO_2_ environment. For HSV-1 and VSV infection, HeLa cells were infected with HSV-1 (MOI = 2), VSV(MOI = 1). After 1 hour, the cells were washed with PBS (Spark Jade) and cultured for an additional 12 h.

### Mice

The Sec10^fl/+^ mice were generated by Cyagen Biosciences through CRISPR/Cas9-mediated gene editing. *Lyz*2-Cre mice were purchased from the GemPharmatech co. ltd. Sec10^fl/+^ mice was crossed with *Lyz*2-Cre mice to obtain *Lyz*2-Cre; Sec10^fl/fl^ mice. All genetic models were on the C57BL/6 background. Mice including both sexes, between the ages of 6–8 weeks were used for all described experiments. Mice genotypes were determined by PCR analysis of tail DNA, and the genotyping primers are as follows (wild-type Sec10 allele is of 133 bp; floxed allele is of 202 bp; positive *Lyz*2-Cre is of 1413 bp): Sec10 forward:5′-CAGTCTTTGGGTTGTGTTTTAAGC-3′, reverse:5′-TCTAAGACACATGTGGTTGGCTTT-3′; *Lyz*2-Cre forward:5′-AGTGCTGAAGTCCATAGATCGG-3′, reverse:5′-GTCACTCACTGCTCCCCTGT-3′.

### Reagents

The proteasome inhibitors MG132, Chloroquine (CQ) and Z-VAD-FMK were purchased from MedChemExpress; poly(I:C) (LMW), poly(dA:dT), and IFN-β were purchased from InvivoGen.

### qRT-PCR

Total cellular RNA was extracted with a Cell Total RNA Isolation Kit (RE − 03111, Foregene, Chengdu, China), eluted in 30 μL of RNase free water and quantified; reverse transcription was performed with 5 × Evo M-MLVRT Master Mix for qRT-PCR (AG11706, AG). SYBR Green based qPCR was performed using 2X SYBR Green Pro Taq HS Premix (AG, China) All real-time PCR values were normalized to β-actin expression to avoid systemic and random errors during sample processing; The sequences of primers are as follows:

h-β-actin-F: 5'-GGAAATCGTGCGTGACAT-3',

R: 5'-AAGGAAGGCTGGAAGAGT-3';

h-IFN-β-F: 5'-GGACAGGATGAACTTTGACA-3',

R: 5'-ACGCCAATCTTCTGGGTGATCT-3';

h-ISG15-F: 5'-GGACCTGACGGTGAAGATGCT-3',

R: 5'-CAGGTGAAATGGCATTTTAGTT-3';

h-ISG54-F: 5'-TATTGGTGGCAGAAGAGGAAGA-3',

R: 5'-CAGGTGAAATGGCATTTTAGTT-3';

h-ISG56-F:5'-TAGACTGTGAGGAAGGATGG-3',

R: 5'-CAGGCGATAGGCAGAGAT-3';

h-STAT1-F:5'-TGGGCTTCATCAGCAAGGAG-3',

R: 5'-GTAGGGTTCAACCGCATGGA-3';

ICP0-F:5'-GGTGTACCTGATAGTGGGCG-3',

R: 5'-GCTGATTGCCCGTCCAGATA-3';

VSV G-F:5'-TGCCCGTCAAGCTCAGATTT-3',

R: 5'-AGCATGACACATCCAACCGT-3';

m-β-actin-F:5'-CCACACCCGCCACCAGTTCG-3',

R: 5'-TACAGCCCGGGGAGCATCGT-3';

m-IFN-β-F: 5'-CAGCTCCAAGAAAGGACGAAC-3',

R: 5'-GGCAGTGTAACTCTTCTGCAT-3′;

m-ISG15-F: 5'-AGAAGCAGATTGCCCAGAAG-3',

R: 5'-TGCGTCAGAAAGACCTCATAGA-3';

m-ISG54-F: 5'-CCTAAACAGTTACTCCACCTTCG-3',

R: 5'-TTGCTGACCTCCTCCATTCT-3′;

m-ISG56-F:5'-TGCTGAGATGGACTGTGAGGAA-3',

R: 5'-TCTTGGCGATAGGCTACGACTG-3'.

### Western blotting and antibodies

Cells were lysed with RIPA lysis buffer (containing ProtLytic Protease and Phosphatase Inhibitor Cocktail (NCM Biotech)) on ice for 30 min. The proteins in the cell lysates were separated by SDS-PAGE and electrotransferred to PVDF membranes, which were blocked for 1 h with 5% nonfat milk, followed by blotting with the indicated antibodies and detection with an Omni-ECL Femto Light Chemiluminescence Kit (Epizyme). The following primary antibodies were used: rabbit monoclonal anti-GFP (catalog No. AB0045), rabbit monoclonal anti-ISG15 (catalog No. CY7086), rabbit monoclonal anti-STAT1(catalog No. CY5861), rabbit monoclonal anti-p-STAT1(Y701) (catalog No.CY5917), rabbit monoclonal anti- STUB1(catalog No. CY8471), rabbit monoclonal anti-β-actin (catalog No. AB0035), mouse monoclonal anti-Myc (catalog No. AB0001), rabbit monoclonal anti-VSV-G (catalog No. AB0053), which were obtained from Abways Technology Inc. mouse monoclonal anti-Sec10 (catalog No. sc-514802), mouse monoclonal anti-ICP0 (catalog No. sc-53070), were obtained from Santa Cruz Biotechnology; mouse monoclonal anti-HA (catalog No. AE008), rabbit monoclonal anti-Flag (catalog No. AE063), which were obtained from ABclonal Technology Inc. The following secondary antibodies were used: horseradish peroxidase (HRP)-conjugated sheep anti-rabbit IgG and HRP-conjugated ECL sheep anti-mouse IgG (Jackson ImmunoResearch Inc., Baltimore, PA, USA). The protein bands were detected with enhanced chemiluminescence (ECL) reagent..

### Gene silencing

Cells were grown until 40% confluence and then incubated with 50 nM Sec10 siRNA (5’-GGUGAAAUCUCCAGAAUGA-3’) or STUB1 (5’- CUGUGAAGGCGCACUUCUU -3’) and Attractene Transfection Reagent (QIAGEN) for 36 h according to the manufacturer’s instructions.

### Enzyme-linked immunosorbent assay (ELISA)

A mouse IFN-β Quantikine ELISA Kit (Multisciences (Lianke), EK2236) was utilized to measure the concentrations of IFN-β in culture supernatants according to the manufacturer’s instructions. Assay Diluent RD1-19 was added to each well, and standard substances, including deionized water, control, or sample, were added to each well. The cells were incubated for 2 h at room temperature. Each well was aspirated and washed, and the process was repeated three times for a total of four washes. Mouse IFN-β Conjugate was added to each well. A new adhesive strip was used to cover the plates, and the plates were incubated for 2 h at room temperature on a shaker. The wash step was repeated. Substrate solution was added to each well and incubated for 30 minutes at room temperature on a benchtop. Stop solution was added to each well. The color in the wells changed from blue to yellow. The optical density of each well was determined within 30 minutes using a microplate reader set to 450 nm. Additionally, the absorbance at a wavelength of 570 nm was also detected, and this value was used to correct for optical imperfections in the plate.

### Dual-luciferase assay

Luciferase reporter assay was performed as previously described [46]. Briefly, HEK293T cells were seeded in a 96-well plate with 2% DMEM and transiently transfected with IFN-β or ISRE luciferase reporter plasmids in combination with Sec10-HA. After 24 h, cells were infection with VSV to stimulate IFN-β or ISRE promoter. Dual-Glo luciferase reporter assay was performed using a dual-luciferase assay kit (Promega Corporation, Madison, WI, USA) and SpectraMax M5 microplate reader (Molecular Devices Instruments Inc., USA).

### Immunofluorescence assay (IFA)

HEK293T cells plated on coverslips in 24-well plates were transfected with the indicated plasmids for 24 h, after which the original culture was discarded. The cells were then washed with PBS, fixed with room-temperature 4% paraformaldehyde (PFA)/PBS for 10 min, washed, permeabilized with 0.1% Triton X-100/TBS for 10 min, and blocked with 5% BSA. Next, the cells were washed and sequentially incubated with primary antibodies at 4°C overnight and secondary antibodies at room temperature for 60 min. The nuclei were stained with DAPI (US Everbright Inc). The secondary antibodies used in this research were Alexa Fluor 350 goat anti-mouse IgG (H + L) and Alexa Fluor 594 goat anti-rabbit IgG (H + L), which were obtained from Invitrogen. Images were acquired using a Leica DMi8 microscope.

### Coimmunoprecipitation (Co-IP)

Co-IP assays were performed using the Pierce Co-IP Kit (Thermo Scientific, Waltham, MA, USA) according to the manufacturer’s protocol. Whole-cell extracts were collected 48 h after transfection and lysed in lysis buffer supplemented with 1 mM PMSF and complete protease inhibitor cocktail on ice for 30 min. After centrifugation for 10 min at 13,000 × g and 4°C, the supernatants were collected and incubated with Protein G Sepharose beads coupled to specific antibodies for 2 h or overnight with rotation at 4°C. The beads were then washed 3× with lysis buffer. The bound proteins were eluted with elution buffer, and the lysates were boiled for 5 min with sample buffer (50 mM Tris-HCl (pH 6.8), 2% SDS, 10% glycerol, 0.1% bromophenol blue and 1% β-mercaptoethanol). The lysates were subjected to immunoblotting analysis with the indicated antibodies.

### TCID_50_ assay

TCID_50_ was determined in HeLa cells infected with 10-fold serially diluted viruses and cultured at 37°C in a 5% CO_2_ atmosphere for 1 h, the cells were washed three times and incubated in serum-free DMEM. Supernatants were collected at 12 hpi and titrated on HeLa cells. TCID_50_ value was calculated using the Reed-Muench method. Each experiment was conducted three times, and each experiment was performed in triplicate.

### Lung histology

Lungs from mock-infected or VSV-infected mice were dissected, fixed in 4% paraformaldehyde, embedded into paraffin, sectioned, stained with hematoxylin and eosin solution, then examined by microscopy (3D HISTECH) for histological changes.

### Statistical analysis

The results are expressed as the mean ± SEM. Comparisons between the different groups were performed by t-test using GraphPad Prism 8. Values *P < 0.05, **P < 0.01, and ***P < 0.001 were considered significant, and ns indicates a nonsignificant difference.

## Supporting information

S1 Fig Depletion of Sec10 promotes the expression of IFN-I and inflammatory cytokines. (A) Immunoblot analysis of Sec10 expression in HeLa cells treated transfected with negative control siRNA (siNC) or Sec10 siRNA (siSec10) for 36 h. (B) HeLa cells were transfected with siNC or siSec10#1 and then treated with poly(I:C) (20 μg/mL) or infected with VSV (MOI = 1) for 12 h, and the transcription levels of *Ifn-β, Isg15*, *Isg54*, and *Isg56* were monitored by qRT-PCR. (C) qRT-PCR analysis of *Ifn-β*, *Isg15*, *Isg54*, and *Isg56* in Sec10^*fl/fl*^ and *Lyz*2-Cre;Sec10^*fl/fl*^ PMs or BMDMs treated with poly(I:C) (20 μg/mL) or poly(dA:dT) (20 μg/mL) for 12 h. (D) ELISA quantification of IFNβ secretion in HeLa cells treated as in (C). (E) qRT-PCR analysis of *Ifn-β, Isg15*, *Isg54*, and *Isg56* mRNA in the PMs or BMDMs from Sec10^*fl/fl*^ and *Lyz*2-Cre;Sec10^*fl/fl*^ mice infected with VSV as indicated time. (F) ELISA quantification of IFNβ secretion in HeLa cells treated as in (E). (G and H) qRT-PCR analysis of *Tnf-α* and *IL-6* mRNA in the PMs or BMDMs from Sec10^*fl/fl*^ and *Lyz*2-Cre;Sec10^*fl/fl*^ mice treated with poly(I:C) (20 μg/mL), poly(dA:dT) (20 μg/mL) or infected with VSV (MOI = 1), HSV-1(MOI = 2). Data are presented as the means ± SEM of three independent experiments.
(TIF)

S2 Fig Sec10 degrades STAT1 and inhibits IFN signaling. (A) qRT-PCR analysis of *Isg15*, *Isg54*, and *Isg56* mRNA expression in the PMs or BMDMs from Sec10^*fl/fl*^ and *Lyz*2-Cre;Sec10^*fl/fl*^ mice treated with IFNβ (20 ng/mL) for 12 h. (B and C) Immunoblot assays of p-STAT1 and STAT1 in the PMs or BMDMs from Sec10^*fl/fl*^ and *Lyz*2-Cre;Sec10^*fl/fl*^ mice treated with poly(I:C) (20 μg/mL), poly(dA:dT) (20 μg/mL) or infected with VSV (MOI = 1), HSV-1(MOI = 2). Data are presented as the means ± SEM of three independent experiments.
(TIF)

S3 Fig Sec10 promotes the K6-linked polyubiquitination of STAT1 at K240 and K652. (A) HEK293T cells transiently cotransfected with Myc-Sec10 and K6-Ub or K6R-Ub for 48 h, the protein lysates were subjected to IP analysis with anti-STAT1 antibody, followed by IB analysis using antibodies as indicated. (B) IP analysis lysates from STAT1-KO HEK293T cells transiently cotransfected with HA-Ub, Myc-Sec10, along with Flag-STAT1 or Flag-STAT1 K240/652R mutant. (C) Immunoblot analysis of extracts from STAT1-KO HEK293T cells transfected with Flag-STAT1 or Flag-STAT1 K240/652R mutant, together with Sec10-HA plasmid.
(TIF)

S4 Fig Sec10 interacts with STAT1 and STUB1. (A and B) Coimmunoprecipitation of endogenous STUB1 with endogenous Sec10 and STAT1 from mouse PMs or BMDMs treated with MG132 and infected with VSV (MOI = 1) for the indicated times.
(TIF)
