# Supplementary figures and images for: Sec10 suppresses antiviral innate immune response by facilitating STUB1-mediated STAT1 degradation
Source: PLoS Pathog. 2025 Sep 8;21(9):e1013472. doi: 10.1371/journal.ppat.1013472 (PMC12425391; doi:10.1371/journal.ppat.1013472)

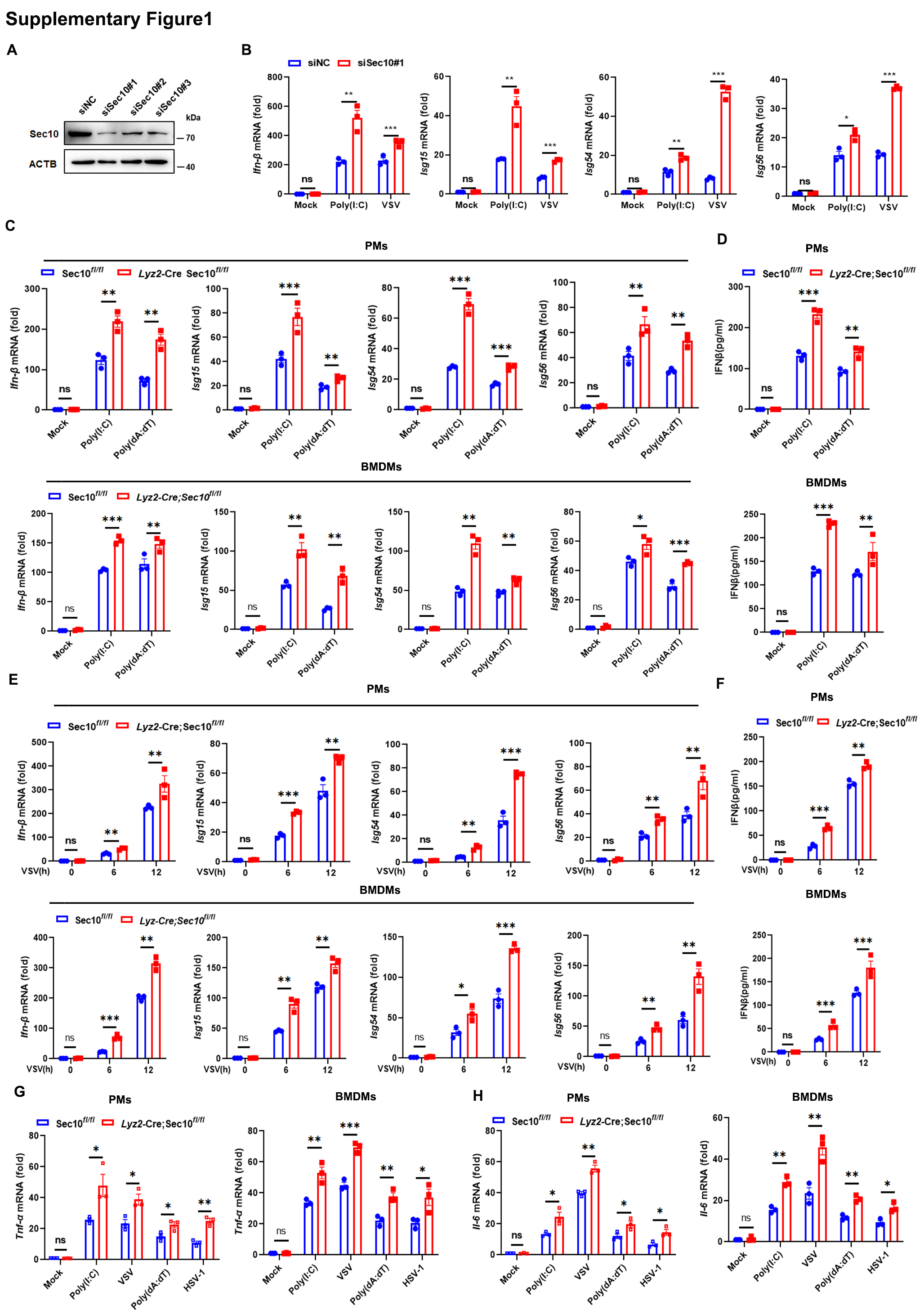

Supplement: S1 Fig — (A) Immunoblot analysis of Sec10 expression in HeLa cells treated transfected with negative control siRNA (siNC) or Sec10 siRNA (siSec10) for 36 h. (B) HeLa cells were transfected with siNC or siSec10#1 and then treated with poly(I:C) (20 μg/mL) or infected with VSV (MOI = 1) for 12 h, and the transcription levels of Ifn-β, Isg15, Isg54, and Isg56 were monitored by qRT-PCR. (C) qRT-PCR analysis of Ifn-β, Isg15, Isg54, and Isg56 in Sec10fl/fl and Lyz2-Cre;Sec10fl/fl PMs or BMDMs treated with poly(I:C) (20 μg/mL) or poly(dA:dT) (20 μg/mL) for 12 h. (D) ELISA quantification of IFNβ secretion in HeLa cells treated as in (C). (E) qRT-PCR analysis of Ifn-β, Isg15, Isg54, and Isg56 mRNA in the PMs or BMDMs from Sec10fl/fl and Lyz2-Cre;Sec10fl/fl mice infected with VSV as indicated time. (F) ELISA quantification of IFNβ secretion in HeLa cells treated as in (E). (G and H) qRT-PCR analysis of Tnf-α and IL-6 mRNA in the PMs or BMDMs from Sec10fl/fl and Lyz2-Cre;Sec10fl/fl mice treated with poly(I:C) (20 μg/mL), poly(dA:dT) (20 μg/mL) or infected with VSV (MOI = 1), HSV-1(MOI = 2). Data are presented as the means ± SEM of three independent experiments. (TIF) [file ppat.1013472.s001.tif]

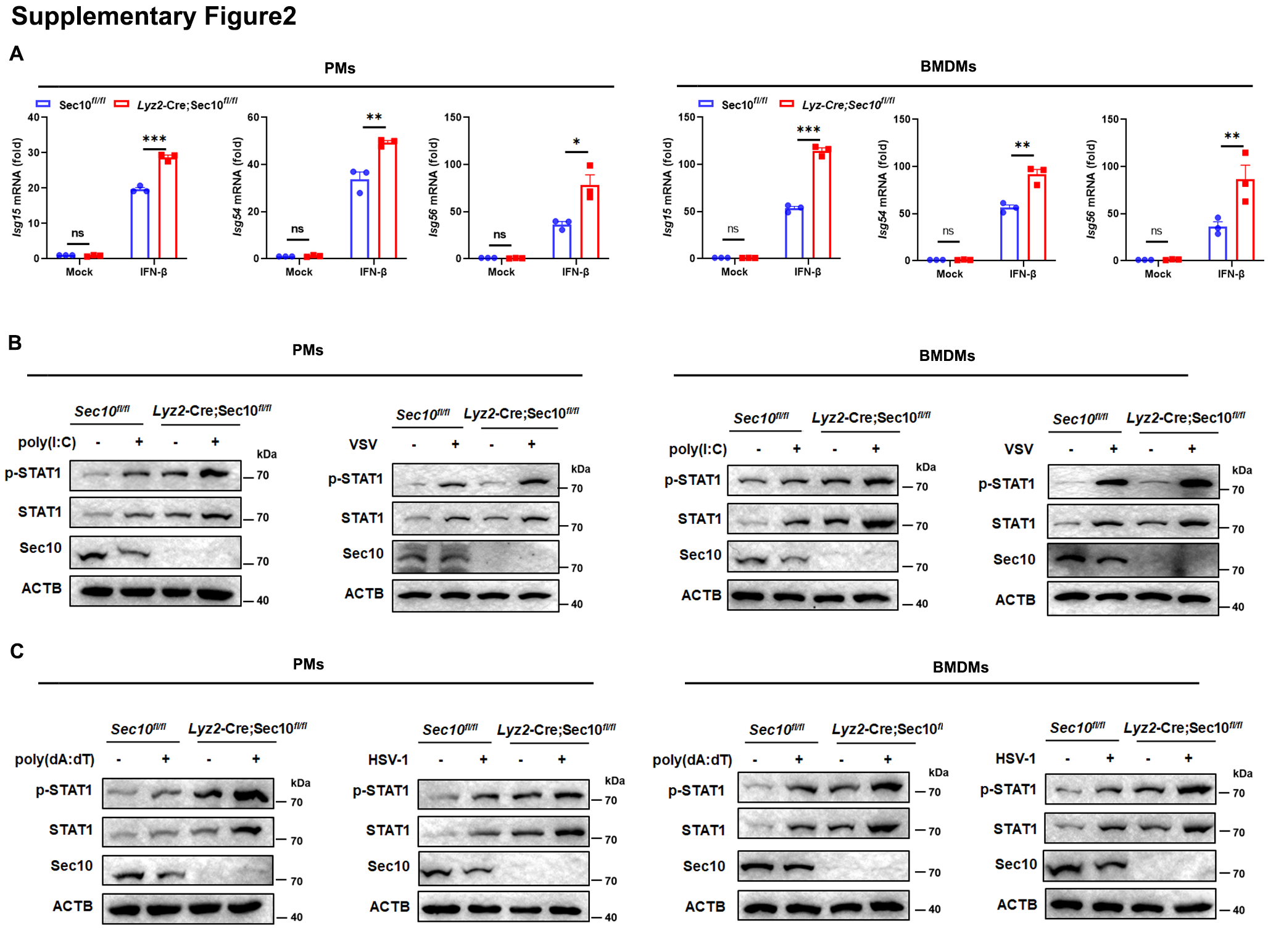

Supplement: S2 Fig — (A) qRT-PCR analysis of Isg15, Isg54, and Isg56 mRNA expression in the PMs or BMDMs from Sec10fl/fl and Lyz2-Cre;Sec10fl/fl mice treated with IFNβ (20 ng/mL) for 12 h. (B and C) Immunoblot assays of p-STAT1 and STAT1 in the PMs or BMDMs from Sec10fl/fl and Lyz2-Cre;Sec10fl/fl mice treated with poly(I:C) (20 μg/mL), poly(dA:dT) (20 μg/mL) or infected with VSV (MOI = 1), HSV-1(MOI = 2). Data are presented as the means ± SEM of three independent experiments. (TIF) [file ppat.1013472.s002.tif]

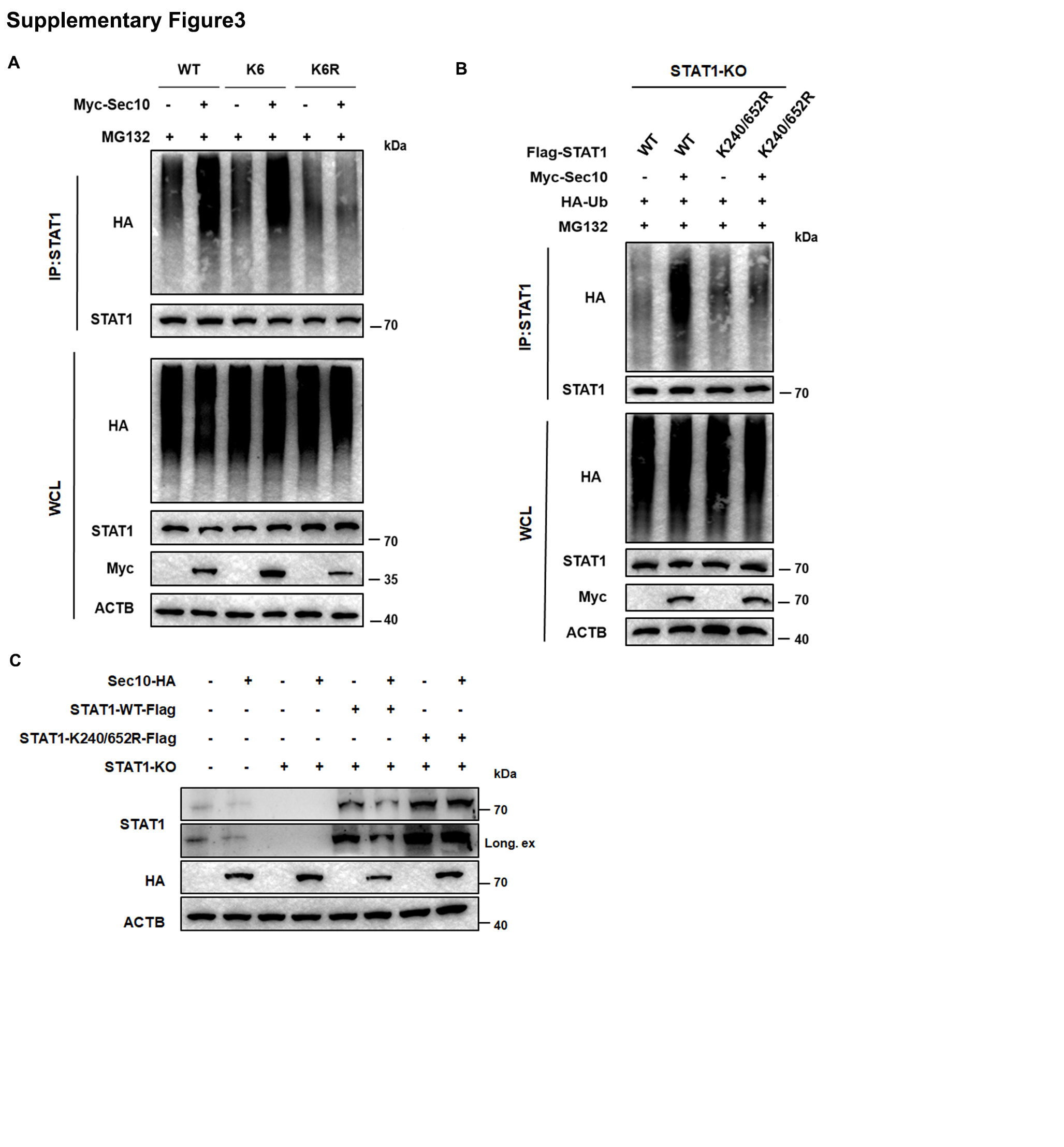

Supplement: S3 Fig — (A) HEK293T cells transiently cotransfected with Myc-Sec10 and K6-Ub or K6R-Ub for 48 h, the protein lysates were subjected to IP analysis with anti-STAT1 antibody, followed by IB analysis using antibodies as indicated. (B) IP analysis lysates from STAT1-KO HEK293T cells transiently cotransfected with HA-Ub, Myc-Sec10, along with Flag-STAT1 or Flag-STAT1 K240/652R mutant. (C) Immunoblot analysis of extracts from STAT1-KO HEK293T cells transfected with Flag-STAT1 or Flag-STAT1 K240/652R mutant, together with Sec10-HA plasmid. (TIF) [file ppat.1013472.s003.tif]

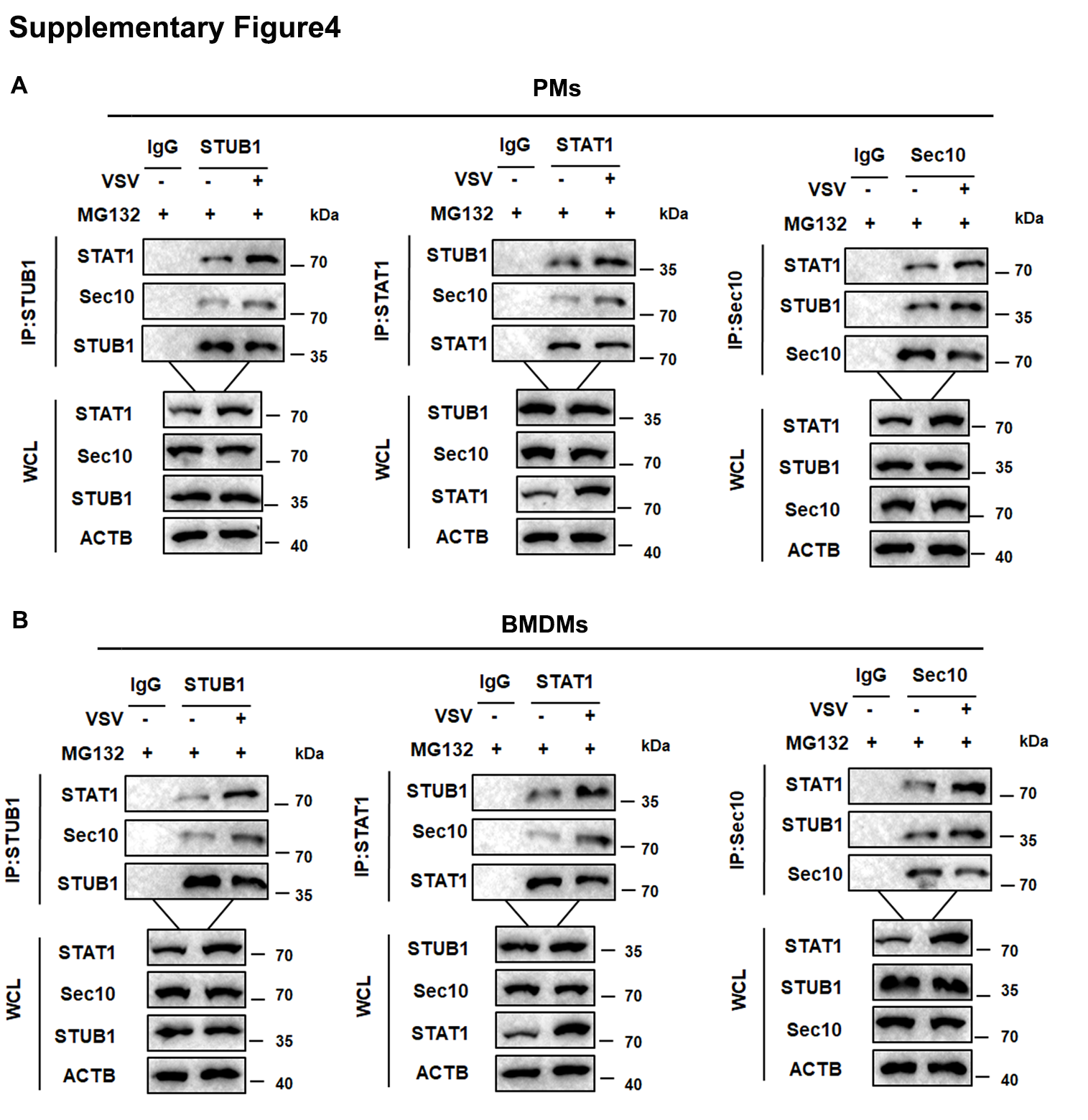

Supplement: S4 Fig — (A and B) Coimmunoprecipitation of endogenous STUB1 with endogenous Sec10 and STAT1 from mouse PMs or BMDMs treated with MG132 and infected with VSV (MOI = 1) for the indicated times. (TIF) [file ppat.1013472.s004.tif]
